# Role of Endoscopic Ultrasound‐guided Gastroenterostomy for Benign Gastric Outlet Obstruction

**DOI:** 10.1002/deo2.70170

**Published:** 2025-07-01

**Authors:** Suprabhat Giri, Saroj Kanta Sahu, Gaurav Khatana, Prasanna Gore, Preetam Nath, Bipadabhanjan Mallick, Jimmy Narayan, Aditya Kale, Sridhar Sundaram

**Affiliations:** ^1^ Department of Gastroenterology & Hepatology Kalinga Institute of Medical Sciences Bhubaneswar India; ^2^ Department of Gastroenterology Medanta‐The Medicity Gurugram India; ^3^ Department of Gastroenterology Renova Neelima Hospital Hyderabad India; ^4^ Department of Gastroenterology Institute of Medical Sciences and Sum Hospital Bhubaneswar India; ^5^ Department of Digestive Diseases and Clinical Nutrition Advanced Centre for Treatment, Research and Education in Cancer Mumbai India; ^6^ Department of Digestive Diseases and Clinical Nutrition Tata Memorial Hospital Mumbai India

**Keywords:** duodenal stricture, endoscopic ultrasound, gastric outlet obstruction, gastroenterostomy, gastrojejunostomy

## Abstract

Benign gastric outlet obstruction (GOO) often results from intrinsic conditions like peptic strictures, caustic‐induced stricture, and surgical anastomoses, and extrinsic conditions like pancreatitis, hematoma, and superior mesenteric artery syndrome. While traditional management involved surgery or endoscopic balloon dilation, endoscopic ultrasound‐guided gastroenterostomy (EUS‐GE) has emerged as a minimally invasive alternative using lumen‐apposing metal stents (LAMS). We aimed to summarize the currently available literature on EUS‐GE in treating benign GOO. EUS‐GE demonstrates high technical success rates, ranging from 95% to 100%, and significant clinical success, typically exceeding 80% in patients with benign GOO. It offers advantages by bypassing the obstruction and potentially providing longer‐lasting relief compared to enteral stenting without the morbidity of surgery. Furthermore, it can serve as a bridge to definitive treatment, allowing for nutritional optimization before surgery or resolution of the underlying condition with subsequent stent removal in a notable proportion of patients. Despite the high efficacy, EUS‐GE is associated with multiple adverse events like maldeployment, bleeding, and ascites with or without infection. Thus, EUS‐GE is a promising and effective minimally invasive modality for managing benign GOO, particularly in patients who fail conventional endoscopic therapies or are poor surgical candidates. However, current evidence is limited by the retrospective nature of many studies, small sample sizes, and the need for longer‐term follow‐up to assess stent durability and the optimal management of indwelling LAMS. Larger prospective studies are warranted to further define the role of EUS‐GE in benign GOO and compare it with other treatment strategies.

## 1 Introduction

Gastric outlet obstruction (GOO), characterized by the mechanical or functional impairment of gastric emptying, can occur due to various malignant and benign etiologies [1]. Although malignancies account for the majority of the cases of GOO, benign etiologies still remain a significant contributor, with peptic ulcer disease (PUD) and chronic pancreatitis (CP) being the most common causes [1]. Traditionally, surgical gastroenterostomy (SGE) has been the gold standard for the management of benign GOO. However, surgical interventions carry inherent risks, including wound infections, anastomotic leaks, and prolonged recovery, increasing morbidity [2, 3].

Endoscopic management offers a minimally invasive alternative to SGE for the management of benign GOO. These include endoscopic balloon dilatation (EBD), enteral stenting (ES) with self‐expanding metal stents (SEMS) or lumen‐apposing metal stents (LAMS), and endoscopic ultrasound‐guided gastroenterostomy (EUS‐GE) [1, 2, 3, 4]. This technique potentially reduces morbidity and mortality compared to traditional surgery, particularly in patients with significant comorbidities or those deemed high‐risk surgical candidates. However, strictures due to corrosives, medications, and CP have lower response rates to EBD [1, 5]. Similarly, there is limited data on ES for benign GOO with high rates of migration, which led to exploring EUS‐GE as an alternative to these modalities [6, 7].

EUS‐GE, in benign GOO, can be offered after the failure of EBD, ES, or both. EUS‐GE allows real‐time visualization of the gastrointestinal tract, enabling the creation of a direct anastomosis between the stomach and duodenum/jejunum utilizing specialized stents like LAMS [2, 8]. The advantages of EUS‐GE are manifold. Its minimally invasive nature translates to reduced pain, shorter hospital stays, faster recovery compared to surgery, and can be performed in those unfit for surgery [1, 2, 9]. As the majority of data on the outcome of EUS‐GE for GOO are reported for malignant etiologies [9, 10], the present review aims to review the current literature on the efficacy and safety of EUS‐GE for benign etiologies.

## 2 Technical Aspects of EUS‐GE

In EUS‐GE, gastro‐enteric anastomosis can be performed through two routes: one is EUS‐gastroduodenostomy, by puncturing the third or fourth portion of the duodenum, and the other is gastrojejunostomy, which targets the proximal jejunum [11]. The small intestinal tract intended for stent deployment should be in close apposition (≤ 1 cm) to the stomach. The contraindication to performing EUS‐GE is the presence of a large amount of ascites, uncorrectable coagulopathy, severe thrombocytopenia, and severe cardiopulmonary disease [12]. Prophylactic antibiotics are administered preprocedural, and in patients with concomitant ascites, they are to be continued for a few days after the procedure to prevent infectious complications [13].

### 2.1 Choice of Stent

LAMS features wide diameters and flanges on both ends, minimizing the migration risk. The Axios stent was introduced in 2012 and is most widely used for EUS‐guided drainage of pancreatic fluid collections [8, 14]. Apart from EUS‐GE, these specialized stents are also used for EUS‐guided biliary drainage and gallbladder drainage, drainage of postsurgical fluid collections, and EUS‐directed transgastric ERCP (EDGE procedure) [15, 16]. Historically, non‐cautery‐assisted LAMS (cold‐LAMS) were used for anastomosis. However, present guidelines recommend using electrocautery‐enhanced LAMS (EC‐LAMS) for EUS‐GE [13], which include the Hot‐Axios stent (Boston Scientific Corp., Marlborough, MA, USA), the Niti‐S Hot‐Spaxus (Taewoong Medical Co., Busan, Republic of Korea), and the HANAROSTENT Hot Plumber Z‐EUS IT (M.I.Tech, Seoul, Republic of Korea) [17, 18]. Table 1 shows the comparison of the three types of EC‐LAMS used for EUS‐GE. EC‐LAMS allows puncture, tract dilation, and stent deployment in a single step without the use of a guidewire, thus simplifying the procedure and reducing the risk of complications. Some prefer to dilate the LAMS with a CRE balloon to achieve the maximum diameter immediately after the procedure. LAMS with wide diameters, e.g., 15 and 20 mm, are preferred to optimize the smooth passage of food [15, 17]. A study comparing 15 mm and 20 mm LAMS reported a significantly higher proportion of patients tolerating soft solid/complete diet in the 20‐mm group (91.2 % vs. 81.2 % with 15 mm, *p* = 0.04).[19] A subsequent meta‐analysis reported no difference in clinical success but a significantly lower reintervention rate with a 20‐mm stent (3.5% vs. 10.3%) [20]. Thus, LAMS with 20 mm should be preferred whenever available due to higher full‐diet tolerability and lower reintervention rates.

**TABLE 1:** Comparison of various electrocautery‐enhanced lumen‐apposing metal stents used for endoscopic ultrasound‐guided gastroenterostomy.

Technical specifications	Hot‐Axios stent (Boston Scientific Corp., Marlborough, MA, USA)	Niti‐S Hot‐Spaxus (Taewoong Medical Co., Busan, Republic of Korea)	HANAROSTENT Hot Plumber Z‐EUS IT (M. I. Tech, Seoul, Republic of Korea)
	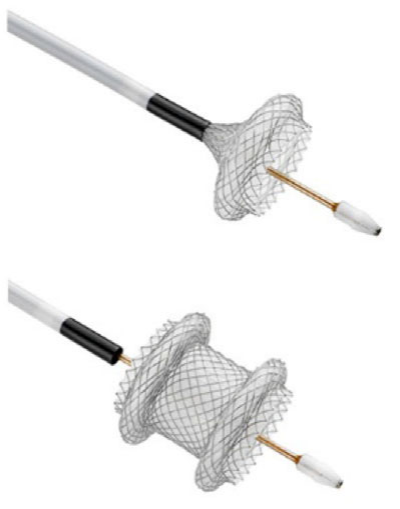	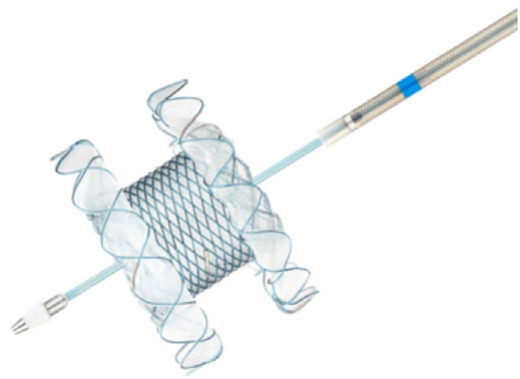	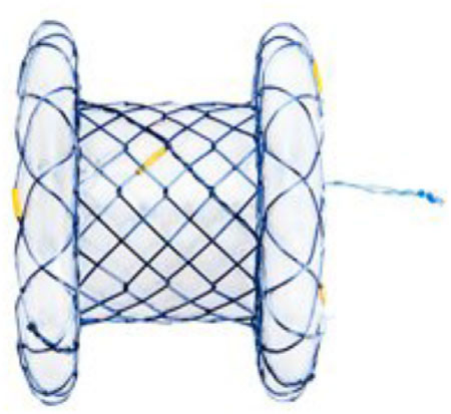
**Flange diameter (mm)**	21, 24, 29	23, 25, 31	22‐28
**Length (mm)**	10	20	20, 30, 40
**Lumen diameter (mm)**	15, 20	16	14, 16
**Delivery catheter (Fr)**	10.8	10	10.5
**Material**	Nitinol‐braided, silicone‐covered	Nitinol, silicone‐covered	Nitinol, silicone‐covered
**One‐handed operation**	Yes	No	Yes

### 2.2 Type of EUS‐GE Technique

**FIGURE 1:**
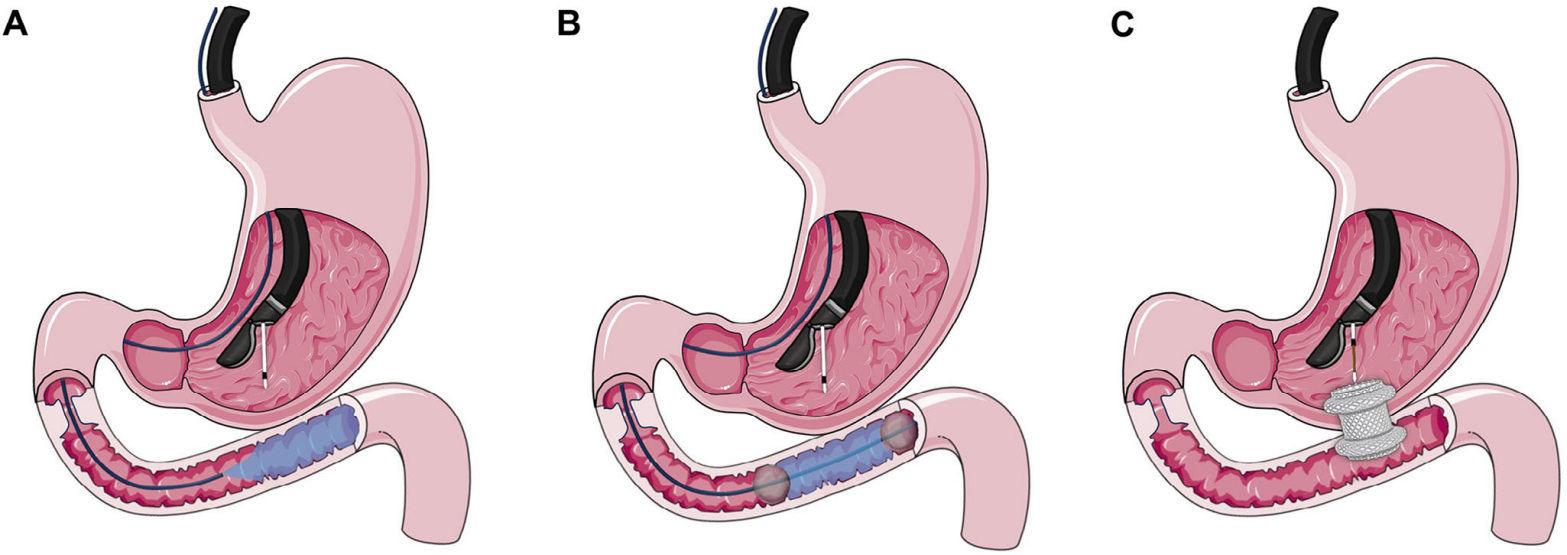
The two most common methods currently used for puncture during endoscopic ultrasound‐guided gastroenterostomy (EUS‐GE): (A) Wireless endoscopic simplified technique using oroenteric catheter and (B) EUS‐guided double‐balloon‐occluded gastrojejunostomy bypass (EPASS), followed by (C) lumen apposing metal stent placement.

EUS‐GE can be performed using either the direct technique or any of the assisted techniques. The assisted techniques require additional devices (e.g., ultraslim endoscope, oroenteric catheter [OEC], and balloons) for small bowel distention and stent placement (Figure 1) [11, 13]. Previously, EUS‐GE was done after puncturing the bowel loop with a needle, followed by guidewire placement and deployment of the stent over the guidewire. However, guidewire placement carries the risk of pushing the small bowel away from the stomach. With the advent of EC‐LAMS, presently, the bowel loop is directly punctured with EC‐LAMS under EUS guidance, followed by stent deployment.

#### 2.2.1 Wireless Endoscopic Simplified Technique

Wireless endoscopic simplified technique (WEST) involves advancing an OEC (typically 7 or 8 Fr) over a guidewire across the site of obstruction into the proximal jejunum. The linear echoendoscope is then inserted alongside the OEC, and sterile water or dye‐mixed saline is infused through the OEC to fill and distend the small‐bowel lumen, creating a clear target for EUS‐guided puncture. Antispasmodics like glucagon and hyoscine allow better visualization by reducing peristalsis and allowing fluid to remain in place for a longer duration [13]. This method is reported to overcome the challenges associated with balloon catheter insertion, such as gastric looping and catheter flaccidity, reducing procedural duration. Nguyen et al. reported a mean procedural duration of 36 min with WEST EUS‐GE [21], which was significantly lower than that reported for the balloon‐assisted techniques (96 min by Khasab et al.) [22]. Another study on EUS‐GE predominant malignant etiologies reported a higher technical success and lower adverse event (AE) rate with the WEST compared to the over‐the‐wire technique [23]. However, there are no comparative studies demonstrating the same in benign etiologies.

#### 2.2.2 EUS‐guided Double‐balloon‐occluded Gastrojejunostomy Bypass

EUS‐guided double‐balloon‐occluded gastrojejunostomy bypass (EPASS) is an assisted method that involves the insertion of a specialized double‐balloon enteric tube over a guidewire that has been passed across the obstruction into the small bowel. The enteric tube in the double balloon is porous, which helps to distend the jejunum (in between the distended balloons) with dye‐mixed saline. This segment of distended bowel loop between the balloons can be targeted directly with EC‐LAMS, followed by stent placement [26]. Other assisted techniques, e.g., single balloon catheter or ultraslim scope‐assisted EUS‐GE, have almost completely been abandoned.

#### 2.2.3 Direct EUS‐GE

A less frequently employed method is the direct needle puncture technique, utilized when neither the endoscope nor the guidewire can negotiate the obstruction. It can also be used to access a targeted bowel limb in altered anatomy and facilitate ERCP in patients with altered foregut anatomy. The echoendoscope is positioned in the stomach, and the small bowel (a collapsed loop) is punctured with a 19‐gauge needle. The small bowel is distended through the needle with dye‐mixed saline, and an antispasmodic is administered. The last step was the placement of an electrocautery‐enhanced LAMS (20 mm size) using the freehand method. The direct needle‐puncture technique is useful in both benign (postsurgical inflammation causing gastric outlet obstruction, pancreaticobiliary limb access in a duodenal switch) and malignant GOO [27, 28, 29]. However, this technique is much more demanding and should only be attempted by experts with extensive experience in EUS‐GE and its salvage approaches.

## 3 Benign Indications of EUS‐GE

The classical indication of EUS‐GE is the palliative treatment for malignant GOO. Of late, there has been an increase in the use of EUS‐GE for benign indications. Table 2 summarizes the indications of benign GOO for which EUS‐GE has been used. Table 3 summarizes the available studies on the role of EUS‐GE in benign etiologies [22, 24, 27, 30, 31, 32, 33, 34, 35, 36, 37, 38, 39, 40, 41]. EUS‐GE combines the immediate effect of stents and the long‐term efficacy of gastroenterostomy and is indicated if the site of obstruction is in the antropyloric area or duodenum (up to the 3rd part). The candidates for EUS‐GE are cases of benign GOO who do not respond to conservative treatment, have persistent malnutrition, and patients who are too frail to undergo SGE. EUS‐GE is also a prospective substitute for ES [1]. Regardless of the etiology and size of the stenosis, symptomatic GOO is considered to be a candidate for EUS‐GE. It is also indicated for patients with afferent loop syndrome [41]. The decision about which treatment to perform is complex and should be individualized, considering patient characteristics, the location and type of obstruction, the presence of ascites, and the experience of the endoscopic team. Patients must be informed of all available options, including SGE and endoscopic alternatives like ES or venting gastrostomy, as applicable.

**TABLE 2:** Benign indications of gastric outlet obstruction.

Intrinsic	Extrinsic
Peptic ulcer disease Caustic ingestion NSAID‐induced strictures Anastomotic stricture Crohn's disease Gastroduodenal tuberculosis Radiation Afferent loop syndrome	Duodenal hematoma Chronic pancreatitis/ groove pancreatitis Acute pancreatitis SMA syndrome Peripancreatic fluid collection Afferent loop syndrome

Abbreviations: NSAID: nonsteroidal anti‐inflammatory drugs; SMA: superior mesenteric artery.

**TABLE 3:** Details of the studies on the role of endoscopic ultrasound‐guided gastroenterostomy for benign indications.

Author, year, study design	No. of patients	Age, in years/male	Indications	Type of procedure/ anastomosis/ stent	Outcomes	Procedural duration, in min	Follow‐up, in days
Khashab, 2015 [22], multicenter, retrospective	7	43–55/ 71.4%	CP: 3, peptic stricture: 2, duodenal hematoma: 1, SMA syndrome: 1	Balloon‐assisted: 7; GJ: 4, GD: 3; 15 mm	Clinical success: 100% GOOSS grade 3–4: 6, grade 2: 1 No recurrence	–	–
Tyberg, 2016 [27], multicenter, prospective	9	–	CP or pyloric stenosis	–	Clinical success: 78%, no recurrence	–	–
Chen, 2018 [24], multicenter, retrospective	26	57.7 ± 13.9/53.8%	CP: 11, surgical anastomosis: 6, peptic ulcer disease: 5, acute pancreatitis: 1, SMA syndrome: 1, caustic injury: 1, and hematoma: 1	Direct: 15, Balloon‐assisted: 7, EPASS: 4; GJ: 21, GD: 5; 15 mm	Clinical success: 84.0%, mean time to oral intake: 1.4 ± 1.9 days, reintervention: 4.8 %, maldeployed stents: 2 and 1 gastric leak needing surgical intervention following elective stent removal	44.6 ± 26.1	176.5 (IQR: 47–445.75)
Chavan, 2019 [30], case report	1	45/male	CP with splenic vein thrombosis with failed endoscopic dilatation for duodenal stricture	EPASS: 1; GJ: 1; 20 mm	Hemoperitoneum—managed conservatively; clinical success on follow‐up		2
Kerdsirichairat, 2019 [31], multicenter, retrospective	9	–	Peptic stricture: 2, surgical anastomosis: 2, SMA syndrome: 1, Crohn's stricture: 1, severe adhesion: 1, intramural duodenal hematoma from acute pancreatitis: 1	Direct: 9; 15 mm	Technical success: 88.9%, clinical success: 77.8%	–	319.5 (IQR 168.8–598)
James, 2020 [32], single‐center, retrospective	22	54.2 ± 13.4/59.1%	Peptic stricture: 5, CP: 5, surgical anastomosis: 4, duodenal hematoma: 3, acute pancreatitis: 1, PFC: 4	Direct: 9, Balloon‐assisted: 8, Saline injection: 5; GJ: 16, GD: 5; 15 mm: 16, 20 mm: 5	Technical success: 100%, clinical success: 95.4%, AE: 19%, severe AE: 14.3%, reintervention: 23.8%, LAMS removal: 18/21 (85.7%) after a mean dwell time of 270 ± 273 days → 15 maintained	66 ± 33.5	465.5 (82–1263)
Havre, 2021 [33], single‐center, retrospective	5	60‐80/‐	CP: 3, Antroduodenal syndrome: 2	Free‐hand: 5; GJ: 5; 15 or 20 mm	Technical success: 100%, clinical success: 100%, AE: 20%, elective change of LAMS after 2.5 years: 1	–	140–945
Nguyen, 2021 [21], single‐center, retrospective	5	–	Peptic stricture: 3, groove pancreatitis: 1, SMA syndrome: 1	Free‐hand: 5	Technical success: 100%	–	–
Sobani, 2021 [34], single‐center, retrospective	8	44.7 ± 19.5/50%	Peptic stricture: 4, CP: 2, SMA syndrome: 2	Free‐hand; GJ; 20 mm	Technical success: 100%, clinical success: 100%, AE: 12.5%, no recurrence of GOO	–	291.7 ± 211.4
Abel, 2024 [35], single‐center, retrospective	18	63 (40–93)/61.1%	Peptic stricture: 6, anastomotic: 1, radiation: 1, pancreatitis: 6, SMA syndrome: 3, hematoma: 1	WEST: 15, retrograde: 3; GJ: 15 or 20 mm	Technical success: 100%, clinical success: 94%, AE: 16.7%, stent removed: 4 (22.2%)	–	286 (88–1444)
Dhar, 2024 [36], case report	1	23/male	Tuberculosis	Direct; GJ; 15 mm	Clinical success on follow‐up, stent removal at 4 months	–	730
Kahaleh, 2024 [37], multicenter, retrospective	31	581 ± 6.2/ 61%	–	Direct or balloon or catheter‐wire assisted; GJ: 31; 15 mm: 10, 20 mm: 21	Technical success: 80.6%, clinical success: 77.4%, maldeployment: 16%, periprocedural AE: 6.7%	104 (20–270)	192
Martinez, 2024 [38], multicenter, retrospective	16	–	CP: 7, Crohn's disease: 2, others: 7	Direct or WEST; GJ; ‐	Technical success: 80.6%, clinical success: 75%, AE: 18.6%	–	–
Wannhoff, 2024 [39], multicenter, retrospective	39	55 (27–76)/61.5%	Acute necrotizing pancreatitis: 39	Direct/ WEST or guidewire‐assisted; GJ: 39; 10, 15, or 20 mm	Technical success: 92.3%, clinical success: 87.2%, maldeployment: 16%, periprocedural AE: 6.7%	58 (19–117)	90
Gonzalez, 2025 [40], single‐center, retrospective	34	53.2 ± 17.4/52.9%	CP: 9, gastroparesis: 16	Direct: 13, WEST: 21; GJ: 34; 20 mm	Technical success: 94.1%, clinical success: 94.1%, AE: 17.6%, maldeployment: 8.8%,	–	90
Trieu, 2025 [41], single‐center, retrospective	90	56.3 ± 15.1/46.7%	Peptic stricture: 21, access for ERCP 17, pancreatitis: 13, post‐surgical: 12, PFC: 10, SMA syndrome: 4, hematoma: 3, others: 10	Balloon or catheter‐wire assisted; GJ: 90; 15 or 20 mm	Technical success: 96.7%, clinical success for GOO: 95.6%, maldeployment: 1.1%, periprocedural AE: 13%, late AE: 5.5%, reintervention: 5.6%, stent removed: 47.8%, stent exchanged: 22.2%	–	666 ± 653.2

Abbreviations: AE: adverse event; CP: chronic pancreatitis; EPASS: endoscopic ultrasonography‐guided double‐balloon‐occluded gastrojejunostomy bypass; ERCP: endoscopic retrograde cholangiopancreatography; GD: gastroduodenostomy; GJ: gastrojejunostomy; GOO: gastric outlet obstruction; GOOSS: Gastric outlet obstruction scoring system; LAMS: lumen‐apposing metal stents; PFC: pancreatic fluid collection; SMA: superior mesenteric artery; WEST: wireless simplified endoscopic ultrasound‐guided gastroenterostomy technique.

## 4 Technical and Clinical Success of EUS‐GE in Benign Etiologies

EUS‐GE has the potential to become a first‐line therapy for patients with GOO due to its potential to reduce morbidity. Khashab et al. reported the first‐ever experience of EUS‐GE in 7 patients with benign GOO [22]. Balloon‐assisted EUS‐GE was the technique used in the study. They reported a technical success rate and clinical success rate of 90% and 100%, respectively. The study reported no procedure‐related AEs or recurrence. Chen et al. published a series of patients undergoing EUS‐GE for benign etiologies, the most common being chronic pancreatitis, surgical anastomosis, and peptic ulcer disease. Twenty‐six patients underwent EUS‐GE with technical and clinical success rates of 96% and 84%, respectively. Half of the patients had a LAMS dilatation [24]. In the study by Abel et al., 16 patients underwent EUS‐GJ for benign GOO from 2017 to 2022. About half the GOO was due to extrinsic etiology. Technical and clinical success rates were 100% and 93%, respectively. One‐fourth of patients had their LAMS removed, and 3/4th of them had an extrinsic etiology. This study stressed that the extrinsic etiology of GOO had a relatively favorable prognosis, given that extrinsic causes of GOO are more likely to resolve [35]. Wanhoff et al. used EUS‐GE for the management of GOO exclusively in acute pancreatitis patients, with a technical success rate of 92.3% and a clinical success rate of 94.4% in those with technical success [39]. Most recently, Trieu et al. reported the largest data on EUS‐GE in benign GOO and reported a clinical success rate of 95.6% in those achieving technical success [41, 42]. In the study by Kahaleh et al., EUS‐GJ had comparable efficacy and incidence of AEs between patients with benign and malignant GOO. This is despite the fact that those with benign diseases had a higher incidence of altered midgut anatomy (22.5% vs. 12%, *p* = 0.038), which in turn may affect successful luminal apposition after stent placement [37].

McCarty et al., in their meta‐analysis, analyzed five studies from 2016 to 2019 involving 199 patients. In 21 cases, gastric outlet obstruction was benign. Immediate technical success was 93%, and clinical success was 90% [43]. The most recent meta‐analysis by Canakis et al. on the performance of EUS‐GE in benign GOO included ten studies (181 patients).[44] Chronic and acute pancreatitis, followed by peptic and surgical strictures, were the most common causes of GOO. The mean procedure time was 66 min, with a 95% technical success rate and 90.6% clinical success rate. Thus, the overall technical success rate of EUS‐GE in benign indications varied from 80.6% to 100%, and the clinical success rate varied from 75% to 100%.

## 5 Safety of EUS‐GE in Benign Etiologies

### 5.1 Early AEs

In a recent meta‐analysis, the pooled rate of AEs with EUS‐GE for benign indications was 11%. Various periprocedural (intra‐ and postprocedural) AEs have been reported following EUS‐GE (Table 4), with the most common and dreaded one being stent maldeployment (SM) [10, 45, 46]. SM is not an unusual AE, occurring in 9.5% of reported cases [47]. SM is classified into four types [46]. Type I: distal flange deployment in the peritoneum and proximal flange in the stomach without indication of an ensuing enterotomy; Type II: deployment of the distal flange in the peritoneum and proximal flange in the stomach despite an enterotomy (i.e., visual confirmation of stent penetration of the targeted small bowel under EUS or fluoroscopy, but migration out on deployment); Type III: deployment of the distal flange in small bowel and proximal flange in the peritoneum; and Type IV: deployment of the distal flange in the colon and proximal flange in the stomach resulting in a gastrocolic anastomosis. Type I and Type II SM constitute approximately 95% of cases of SM, with Type I being the most common [46].

**TABLE 4:** Details of periprocedural adverse events reported in various studies on endoscopic ultrasound‐guided gastroenterostomy for benign indications.

Author, year	Details of adverse events
Khashab, 2015 [22]	0/7
Chen, 2018 [24]	2/26; Mild: Type I misdeployment (*n =* 2)
Chavan, 2019 [30]	1/1; Moderate: hemoperitoneum (*n =* 1)
Kerdsirichairat, 2019 [31]	1/9; Moderate: hemoperitoneum (*n =* 1)
James, 2020 [32]	4/22; Mild: abdominal pain (*n* = 1), Severe: bleeding (*n* = 1), LAMS migration and impaction (*n* = 1), Type IV misdeployment (*n* =1)
Havre, 2021 [33]	0/5
Sobani, 2021 [34]	1/8; Mild: jejunal ulcer
Abel, 2024 [35]	3/18; Mild: bleeding (*n =* 1), moderate: bleeding (*n =* 2)
Dhar, 2024 [36]	0/1
Kahaleh, 2024 [37]	9/31; Grade I: Infection (*n =* 1), Abdominal pain (*n =* 1), Grade II: Bacterial peritonitis (*n =* 1), Ascites infection (*n =* 1), Grade III: Release into peritoneal cavity, closure with OTSC clip (*n =* 2), GI bleeding (*n* = 1), retroperitoneal abscess (*n =* 1), LAMS‐related jejunal ulcer with bleeding (*n =* 1)
Martinez, 2024 [38]	Intra‐procedural: 18.6% (*n =* 3), post‐procedural 37.5% (*n =* 6)
Wannhoff, 2024 [39]	0/39
Gonzalez, 2025 [40]	Intraoperative: Misdeployment (*n =* 3), postoperative (*n =* 6)
Trieu, 2025 [41]	6/90; bleeding (*n =* 3), misdeployment (*n =* 1), dislodgement (*n =* 1), bacterial peritonitis (*n =* 1)

Abbreviation: LAMS: lumen‐apposing metal stents; OTSC: over‐the‐scope clip.

The causes of SM are inadequate distention of the bowel, too much small bowel mobility, puncture outside the target bowel loop, slippage of the stent during deployment due to poor visualization of the bowel loop, and deployment of the second flange outside the working channel [48, 49]. To decrease this risk, glucagon is given before the puncture of the small intestine with the LAMS in our cases, as opposed to during the distension of the small intestine, which may be the practice of others. Particularly in the direct needle‐puncture technique, the inability to continue to distend the small bowel via a nasojejunal tube can result in a less‐than‐ideal target, increasing the risk of SM. In case of LAMS misdeployment, the stent should be removed, followed by closure of the gastric defect with clips or sutures (For type I and II maldeployment) [50]. However, this is not always required, especially if the salvage LAMS is placed nearby.

Other reported AEs include luminal bleeding, hemoperitoneum, jejunal ulcer, ascites ± bacterial peritonitis, and retroperitoneal abscess. Thus, there is a need for data on the need and duration of periprocedural antibiotics for EUS‐GE. An AE unique to the direct needle‐puncture technique is puncturing and injecting intramurally when targeting a small bowel limb. In addition, a more distal jejunal limb may be inadvertently used to create the anastomosis, which can result in diarrhea and/or malabsorption.

### 5.2 Delayed AEs

Concerning the delayed AEs, Havre et al. reported stent migration in one out of the five patients undergoing EUS‐GE for benign indications [33]. Trieu et al. reported stent occlusion by food (mean interval from procedure to stent occlusio*n =* 36 days) and migration (after 33 days) rates of 2.2% and 1.1%, respectively, on follow‐up after EUS‐GE, with the [41]. Stent occlusion was managed by endoscopic cleaning followed by dietary modification, while migration was managed by removal of LAMS and placement of an esophageal covered stent across the anastomosis. One patient developed a gastrocolic fistula, and two developed stent delamination requiring removal [41]. Six studies have reported on the incidence of reintervention (mean interval varying from 28 to 228 days) due to the recurrence of symptoms, with a pooled rate of 7%. This is in comparison to the pooled rate of stent occlusion of 1% in studies on malignant indications. This difference may be due to the fact that patients with benign etiologies have a prolonged survival with a long duration of stent in place, leading to a higher rate of occlusion/reintervention.

### 5.3 Need for Endoscopic Surveillance

While EUS‐GE with LAMS can serve as a temporizing measure or a bridge to surgery for benign GOO, some patients with chronic benign GOO or those unfit for surgery may require the stent to remain in place long‐term. Long‐term placement may be associated with delayed AE like stent obstruction, migration, delamination, embedding, fistula formation, and delayed perforation, requiring endoscopic surveillance for early diagnosis and management. One study recommended annual stent exchanges in patients requiring long‐term stent placement to potentially prevent stent delamination and occlusion [41]. Another study recommended stent removal as soon as there is evidence of resolution of GOO on imaging or endoscopy [32]. However, due to limited data, the optimal strategy, frequency, and timing of surveillance or prophylactic stent exchange are still areas requiring further investigation.

## 6 Considerations for Stent Removal

### 6.1 Timing of Stent Removal

Stents are often left in place until the underlying condition resolves. However, the duration over which a LAMS can be expected to remain patent and have no tissue ingrowth is yet to be established. Determining the optimal time for stent removal, especially in benign GOO, is crucial. Removing too early may lead to recurrence, while leaving it too long increases the risk of complications like delamination and embedding [41]. In a study on benign GOO, stents remained in place for a median of 286 days (range 88–1444 days), with some patients having stents patent for nearly 4 years [35]. Another study reported a mean dwell time of 270 days for electively removed stents in benign GOO [32]. In cases of acute pancreatitis‐induced GOO, LAMS removal was possible in 18 out of 39 patients, with a mean time to removal of 7.5 months (range 1–44 months). Early EUS‐GE (≤6 weeks) in this context was associated with a shorter time to LAMS removal (3 months) compared to late EUS‐GE (>6 weeks, 11 months), although this was not statistically significant [39]. One study reported annual stent replacements in a patient with benign GOO for 8 years without stent‐related complications [41].

### 6.2 Indications for Stent Removal

The resolution of GOO is a primary reason for LAMS removal. These include resolution of pancreatic inflammation/necrosis/collection, healing of peptic ulcers, resolution of duodenal hematoma, and stricture resolution after serial dilations or surgical treatment. EUS‐GE can serve as a bridge to definitive surgery, and the stent may be removed during the surgical procedure. This allows for improved quality of life and nutritional maintenance before surgery [41]. Stent removal is more likely in patients with extrinsic causes of GOO, as these causes are often reversible. 75% of stent removals in one study were in patients with extrinsic GOO etiologies [35].

### 6.3 AEs Related to Removal

Gastric leak is a severe AE that has been reported to require surgical intervention following elective LAMS removal [24]. After LAMS removal, there is a risk of the original GOO recurring. In one series, three patients experienced recurrence after LAMS removal, with one requiring surgical conversion and two needing placement of a second LAMS [32]. One study reported the incidental detection of a gastrocolic fistula three months after LAMS removal [39]. Thus, it's important to note that while LAMS removal is often performed electively after the resolution of benign GOO, these potential AEs highlight the need for careful patient selection, monitoring, and expertise in managing this procedure. Despite this, the incidence of these AEs related to LAMS removal is rare, as reported from the LAMS registry in a recent study from Spain [51]. The long‐term effects of LAMS indwelling and the safety of its removal continue to be areas of study.

## 7 Comparison of EUS‐GE With Other Modalities for Benign GOO

**FIGURE 2:**
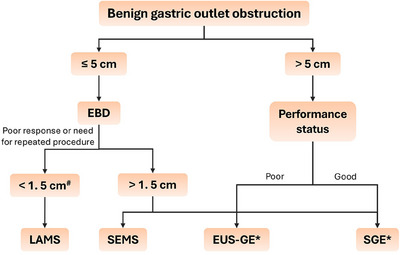
Step‐wise approach to the management of benign gastric outlet obstruction with the current placement of endoscopic ultrasound‐guided gastroenterostomy in the management (EUS‐GE). EBD: endoscopic balloon dilatation; EUS‐GE: endoscopic ultrasound‐guided gastroenterostomy; LAMS: lumen‐apposing metal stent; SEMS: self‐expanding metal stent; SGE: surgical gastroenterostomy. ^#^[3, 53, 54]. *Other factors that need to be considered include reversibility of the condition (EUS‐GE to be preferred in reversible conditions), presence of altered anatomy (EUS‐GE and SGE for altered anatomy), cost, and the availability of expertise.

Multiple studies have compared the outcomes of EUS‐GE with ES and SGE for GOO. A previous meta‐analysis reported comparable rates of technical success (95.2% vs. 96.9%), clinical success (93.3% vs. 85.6%), and AEs (10.7% vs. 19.7%) between EUS‐GE and ES. However, the rate of reintervention was significantly lower with EUS‐GE (4% vs. 23.6%) [52]. A subsequent meta‐analysis reported a higher pooled clinical success rate without recurrence (odds ratio [OR], 5.08; 95% confidence interval [CI], 3.42–7.55) and a similar AE rate (OR 0.57, 95% CI 0.29–1.14) with EUS‐GE compared to ES. On the contrary, EUS‐GE was associated with a similar clinical success rate without recurrence (OR, 1.94; 95% CI, 0.97–3.88) and a lower AE rate (OR 0.17, 95% CI 0.10–0.30) with EUS‐GE compared to SGE [53]. However, most of the studies included in these two meta‐analyses had most patients with GOO due to malignant etiologies. There is limited data comparing the outcomes of EUS‐GE with ES and SGE in benign indications. Martinet et al. reported a significantly higher technical success rate with SGE compared to EUS‐GE (100% vs. 75%, *p* = 0.035). However, the clinical success rate among those having technical success was 100% with EUS‐GE, compared to 78.9% with SGE. This higher clinical success may be attributed to the fact that clinical success was defined as the ability to tolerate oral intake prior to hospital discharge, and patients undergoing SGE will have a prolonged recovery compared to EUS‐GE, resulting in a longer time to oral diet tolerance. The duration of the procedure and hospital stay were also significantly shorter in the EUS‐GE group [38]. In the absence of definitive data comparing the role of EUS‐GE, ES, and SGE in benign etiologies, the optimal treatment modality remains to be decided. The choice of treatment needs to be individualized on a case‐by‐case basis. Figure 2 outlines the management of benign GOO with the current role of EUS‐GE.

## 8 Limitations and Future Perspectives

The studies on EUS‐GE in benign indications are limited by a retrospective study design with a small sample size, which inherently limits the ability to establish causality and may introduce selection bias. Secondly, many studies are conducted at single tertiary referral centers, often with experienced operators, which may limit the applicability of the results to lower‐volume centers or less experienced endoscopists. Third, different studies may employ various techniques for performing EUS‐GE (e.g., direct vs. balloon‐assisted) and utilize LAMS of different sizes (e.g., 15 mm vs. 20 mm), which can affect outcomes. Fourth, there is limited data comparing EUS‐GE and SGE for benign indications, necessitating future research on this topic. There is a paucity of literature on the long‐term management of indwelling LAMS, including the optimal timing for stent exchange or removal, especially in benign conditions where the obstruction might resolve. These limitations highlight the need for more prospective, multicenter, randomized controlled trials with longer follow‐up periods to definitively establish the role of EUS‐GE in the management of benign GOO and to optimize patient selection and procedural techniques.

## 9 Conclusion

EUS‐GE appears to be a promising and increasingly utilized minimally invasive technique for the management of benign GOO, associated with high technical and clinical success rates. EUS‐GE can serve as a temporary measure in benign reversible conditions, allowing for the resolution of the underlying obstruction and the potential removal of the LAMS, thus avoiding permanent surgical intervention in a significant number of cases. However, EUS‐GE is not without its limitations. In light of evolving data, we can become stronger in our recommendations on EUS‐GE for benign GOO, also reflecting the current move far away from surgery. Technical challenges, such as difficulties in identifying a suitable jejunal loop or achieving successful stent deployment, can arise. Potential complications include SM, migration, leakage, bleeding, and peritonitis. Careful patient selection, meticulous technique, and expertise in advanced endoscopy are essential to optimize outcomes and minimize risks. Ongoing research and clinical trials are further refining the procedure, expanding its applicability, and enhancing its long‐term efficacy.

## Ethics Statement

Not applicable for a review article.

## Conflicts of Interest

The authors declare no conflicts of interest.

## Data Availability

The authors have nothing to report.
